# Transcriptomics of Temporal- versus Substrate-Specific Wood Decay in the Brown-Rot Fungus *Fibroporia radiculosa*

**DOI:** 10.3390/jof9101029

**Published:** 2023-10-19

**Authors:** Byoungnam Min, Steven Ahrendt, Anna Lipzen, Cristina E. Toapanta, Robert A. Blanchette, Dan Cullen, David S. Hibbett, Igor V. Grigoriev

**Affiliations:** 1 US Department of Energy Joint Genome Institute, Lawrence Berkeley National Laboratory, Berkeley, CA 94720, USA; mbnmbn00@gmail.com (B.M.);; 2 Department of Plant and Microbial Biology, University of California Berkeley, Berkeley, CA 94720, USA; 3 Department of Plant Pathology, University of Minnesota, St. Paul, MN 55108, USA; 4 USDA Forest Products Laboratory, Madison, WI 53726, USA; 5 Biology Department, Clark University, Worcester, MA 01610, USA

**Keywords:** brown-rot fungi, transcriptomics, wood decay, *Fibroporia radiculosa*

## Abstract

Brown-rot fungi lack many enzymes associated with complete wood degradation, such as lignin-attacking peroxidases, and have developed alternative mechanisms for rapid wood breakdown. To identify the effects of culture conditions and wood substrates on gene expression, we grew *Fibroporia radiculosa* in submerged cultures containing Wiley milled wood (5 days) and solid wood wafers (30 days), using aspen, pine, and spruce as a substrate. The comparative analysis revealed that wood species had a limited effect on the transcriptome: <3% of genes were differentially expressed between different wood species substrates. The comparison between gene expression during growth on milled wood and wood wafer conditions, however, indicated that the genes encoding plant cell wall-degrading enzymes, such as glycoside hydrolases and peptidases, were activated during growth on wood wafers, confirming previous reports. On the other hand, it was shown for the first time that the genes encoding Fenton chemistry enzymes, such as hydroquinone biosynthesis enzymes and oxidoreductases, were activated during submerged growth on ground wood. This illustrates the diversity of wood-decay reactions encoded in fungi and activated at different stages of this process.

## 1. Introduction

Brown-rot basidiomycetes are primary wood decomposers in coniferous forests and play a crucial role in the conversion of plant biomass [1]. The brown-rot decay process selectively leaves modified lignin while removing holocellulose [1]. Many brown-rot fungi occur in at least five independent clades of Agaricomycotina: Polyporales, Boletales, Gloeophyllales, Agaricales, and Dacrymycetales, indicating that they are products of convergent evolution [2]. Also, brown-rot fungi tend to be either generalists or gymnosperm specialists, in contrast to white-rot fungi, which are typically angiosperm specialists [3]. Due to these distinct evolutionary and physiological features, brown-rot fungi have been targets for extensive genomic and transcriptomic studies.

Previous transcriptomic studies of brown-rot basidiomycetes used various substrates, such as glucose, cellulose, ball-milled wood (aspen, pine, and spruce), and wood wafers, with different lignin–glucose ratios [4,5,6,7,8]. Their differential gene expression analyses mainly focused on genes involved in plant cell wall decay and Fenton chemistry. Recently, Zhang et al. [9] implemented a “space-for-time” design in which mycelium of *Postia placenta*, the established model for brown-rot decay studies, grew along a wood wafer, and samples were extracted from the sections at different depths to identify temporally regulated genes involved in the early and late stages of wood decay.

In brown-rot fungi, two transcriptionally regulated decay stages are established: early chemical depolymerization and late enzymatic saccharification [1,9]. In the early stage, the Fenton reaction (H_2_O_2_ + Fe^2+^ → OH^−^ + Fe^3+^ + •OH) generates hydroxyl radicals that attack polysaccharides. However, because those radicals can damage fungal cells, it was hypothesized that fungi secrete oxalates that chelate with Fe^2+^/Fe^3+^ ions to protect nearby hyphae [10]. As the density of oxalate molecules decreases away from the hyphae, the enzymes involved in hydrogen peroxide generation and iron cycling coordinate for the consistent occurrence of Fenton reactions. When hydroxyl radical-based breakdown progresses further, the decay process proceeds to the late stages, in which hydrolytic enzymes are released onto the depolymerized substrates [9].

Here, we explore the temporal- and substrate-specific transcriptome patterns of *Fibroporia radiculosa*, a brown-rot Polyporales fungus tolerant to copper-based wood preservatives. We first compared the transcriptomes of aspen, pine, and spruce wood species to find out how different wood components impact gene expression. Then, we examined gene expression under two distinct culture conditions—intact wood and wood powder for 30 days—to determine whether structure deconstruction influences gene expression.

## 2. Materials and Methods

### 2.1. Culture Conditions and RNA Extraction

The culture of *F. radiculosa* was provided by the USDA Forest Products Laboratory (Madison, WI, USA). The mycelium was cultivated on three types of wood substrates—quaking aspen (*Populus tremuloides*, hardwood), loblolly pine (*Pinus taeda*, softwood), and white spruce (*Picea glauca*, softwood)—under two culture conditions—submerged cultures containing Wiley milled wood (5 days, across all three substrates) and solid-state wood wafers (30 days, exclusive to pine and spruce).

We used the same method for preparing cultures and extracting RNA described in the previous report [8]. For the submerged culture, we used basal salts media with 1.25 g of Wiley-milled wood. Following autoclaving at 121 °C for 15 min, triplicate cultures for each substrate were inoculated with mycelium scraped from malt extract agar (2% [wt/wt] malt extract, 2% [wt/wt] glucose, 0.5% peptone, 1.5% agar) and placed on a rotary shaker (150 rpm) at 22 to 24 °C. Five days after inoculation, mycelia and pellets of various sizes were collected via filtration through Miracloth (EMD Millipore, Burlington, MA, USA) and stored at −80 °C. For solid-state culturing, the soil mixture for the microcosm was prepared using two parts of loam soil, two parts of vermiculite, and one part of peat moss that was wetted to capacity and placed in glass jars (473 mL). Two wood feeder strips corresponding to each type of wood were placed together on top of the soil mixture and autoclaved twice for 1 h at 121 °C, with a 24-hour period in between sterilizations. A 5-millimeter-diameter plug of media containing mycelium was placed at the corner of the feeder strips. When the mycelium covered the feeder stripped completely, thin (10 × 15 × 1 mm), longitudinally cut wood wafers of each type of wood were placed on the feeder strips and incubated at 22 °C. The wood wafers were removed after 30 days, snap-frozen in liquid nitrogen, and stored at −80 °C.

RNA purification was performed using the modified method devised by Miyauchi et al. [11]. Initially, all samples were pulverized under liquid nitrogen using a SPEX 6775 mill (Metuchen, NJ, USA). Subsequently, the samples underwent pretreatment with TRIzol and Chloroform for cell lysis and phase separation. The nucleic acids were then pelleted through centrifugation at 40,000× *g* for 10 min at 4 °C.

### 2.2. RNA-Seq Library Construction

The quantity and quality of the extracted RNA samples were checked using a Qubit fluorometer (Thermo Fisher Scientific, Waltham, MA, USA) and a 2100 Bioanalyzer (Agilent, Santa Clara, CA, USA), respectively. Starting with 1 g of total RNA per sample, mRNA was selected using poly(A) with the TruSeq Stranded mRNA HT sample prep kit (Illumina, San Diego, CA, USA), and plate-based RNA libraries were prepared via the Sciclone NGS robotic liquid handling system (PerkinElmer, Waltham, MA, USA), with eight cycles of PCR used for library amplification. The prepared libraries were quantified using a next-generation sequencing library quantitative PCR kit (KAPA Biosystems, Wilmington, MA, USA) and run on a LightCycler 480 real-time PCR instrument (Roche, Basel, Switzerland). Sequencing was performed on the HiSeq2000 sequencer (Illumina, San Diego, CA, USA) following a 1 × 101 indexed run recipe.

Sequenced reads were filtered for quality. BBDuk (https://sourceforge.net/projects/bbmap/, accessed on 9 October 2018) was used to trim artifact sequences (kmer = 25, one mismatch allowed) and remove RNA spike-in reads, PhiX reads, and reads containing any Ns. Trimming for quality was performed using the Phred trimming method set at Q6. Subsequently, we removed reads < 33 bp. The number of reads and mapping rates are summarized in Supplementary Table S1.

### 2.3. Identification of Differentially Expressed Genes

Filtered reads were mapped to the reference *Fibroporia radiculosa* TFFH 294 genome [12] using HISAT v2.1.0 [13] (-k 1) and counted for each gene using the featureCounts [14] (-s 2 --primary). Only primary hits assigned to the reverse strand were included in the counts. We used DESeq2 v3.10 [15] for the differential gene expression test with raw rather than normalized gene counts. The genes with an adjusted *p* < 0.05 and a fold change > 4 were denoted as differentially expressed.

### 2.4. The Genes Involved in Fenton Reaction

The genes involved in Fenton reaction were annotated using CAZy (Carbohydrate Active Enzymes database, http://www.cazy.org/, accessed on 2 May 2019) [16], Pfam [17], Enzyme Commission [18], and orthologs assigned via OrthoFinder 2.5.4 [19], as described in the previous reports [1,9]. The detailed gene list and annotation method are summarized in Supplementary Table S2. The aryl-alcohol oxidase proteins (AA3_2, AAO—AADH) were aligned using MAFFT v7.429 [20] (--auto) and FastTree v2.1.11 SSE3 (http://www.microbesonline.org/fasttree/, accessed on 3 August 2019) for tree calculation.

### 2.5. Expanded and Contracted Gene Families

The following five genomes of Polyporales brown-rot fungi were used for comparative gene family analysis: *Daedalea quercina*, *Fomitopsis pinicola*, *Laetiporus sulphureus*, *Postia placenta*, and *Wolfiporia cocos* (Supplementary Table S3). We identified orthologs in these genomes using OrthoFinder 2.5.4 [19] with default options. We performed Fisher’s exact test [21] for each gene family to obtain expanded or contracted gene families in *F. radiculosa*. We assigned functional annotation to each gene family in which >20% of family members shared predicted function.

### 2.6. Statistics Analyses

To examine transcriptomic variance between samples, we performed principal component analysis (PCA) using gene read counts. These counts were regularized through logarithmic transformation using DESeq2 v3.10, stabilizing the systematic variance trends. We employed Pearson’s correlation calculation using transformed gene read counts for one-to-one sample comparisons. We performed Fisher’s exact test to compare the number of members in a gene family between *F. radiculosa* and five other brown-rot fungi.

## 3. Results and Discussion

### 3.1. Wood Species Have a Limited Effect on Fungal Transcriptome

*Fibroporia radiculosa* is a brown-rot fungus belonging to Polyporales in the division Basidiomycota. The genome of this fungus was sequenced and annotated before in [12] and offers a reference critical for our transcriptomics analysis. To study differential gene expression at different substrates and stages of *F. radiculosa* growth, the mycelium of the species has been grown on three wood substrates—quaking aspen (*Populus tremuloides*, hardwood), loblolly pine (*Pinus taeda*, softwood), and white spruce (*Picea glauca*, softwood)—and in two culture conditions—submerged cultures containing Wiley milled wood (5 days, on all three substrates) and solid-state wood wafers (30 days, on pine and spruce only). The mycelium did not grow well on the Aspen wafer, so we could not extract a sample for RNA sequencing.

**Figure 1:**
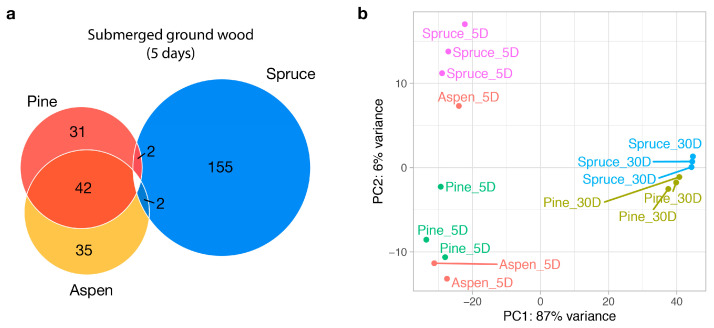
Transcriptomic landscape of *Fibroporia radiculosa*. (**a**) A Venn diagram of differentially expressed genes between substrates in submerged milled wood (5 days). Each number indicates the number of genes that are up-regulated over other conditions. (**b**) Principal component analysis plot with read counts transformed via a regularized logarithm. The labels ‘5D’ and ‘30D’ indicate samples collected at 5 days (submerged wood) and 30 days (wood wafers), respectively.

In the five-day milled wood samples, there were 13–190 differentially expressed genes (adjusted *p* < 0.05 and fold change > 4) between the substrates, i.e., <3% of the total 9262 predicted genes. The aspen and pine expression profiles were almost indistinguishable, with only 13 genes differentially expressed, including genes encoding a cytochrome P450, expansin, transporter, short-chain dehydrogenase, and secreted amine oxidoreductase (Supplementary Data S1). This result was surprising because pine is a softwood, whereas aspen is a hardwood, and different transcriptional responses might be expected during growth on different lignocellulose profiles. We grouped the genes into aspen/pine- and spruce-specific categories, which contained 42 and 152 genes, respectively (Figure 1a). The two groups were functionally distinct in that aspen/pine-specific genes encode oxidoreductases and enzymes involved in fatty acid biosynthesis and the regulation of nitrogen, while spruce-specific genes encode carbohydrate-active enzymes (e.g., glycoside hydrolases, carbohydrate esterases, and lytic polysaccharide mono-oxygenases), cytochrome P450s, lipases, proteases, and sugar transporters. In addition, 64.9% of the up-regulated spruce-specific genes were predicted to be secreted, compared to 35.7% of the up-regulated aspen/pine-specific genes.

Despite their seemingly distinct functions regulated by substrates, it is difficult to conclude that those differentially expressed genes are specifically induced via substrate composition. Firstly, most of those genes did not show differential expression in 30-day samples. In other words, all down-regulated genes in five-day samples ended up being activated in 30-day samples. Secondly, in the principal component analysis plot (Figure 1b), one aspen sample was clustered with spruce samples. From the Pearson’s correlations of aspen and spruce samples, we observed that although the outlier aspen sample has a higher correlation with spruce samples (0.34–0.44 with other aspen samples compared to 0.64–0.83 with spruce samples, Supplementary Figure S1), it is lower than those of intra-spruce samples (0.88–0.96), making it unlikely that it is mislabeled. Thirdly, several inter-substrate comparisons had a higher correlation than intra-substrate comparisons. For example, one of the pine samples showed a higher correlation with spruce (0.96) than the average intra-spruce correlations (0.92). Based on these observations, we suggest that the transcriptomic difference may not be solely derived from the substrate.

Since brown-rot fungi have two degradation modes that are regulated via transcription [9] but hard to separate, we might have captured the samples in the different degradation modes. It has also been reported that degradation activities are similar between softwood and hardwood in a transcriptomic and proteomic study of a white-rot fungus, namely *Pycnoporus coccineus* [22].

### 3.2. Many Genes Involved in Fenton Reaction Are Up-Regulated in Submerged Ground Wood

**Figure 2:**
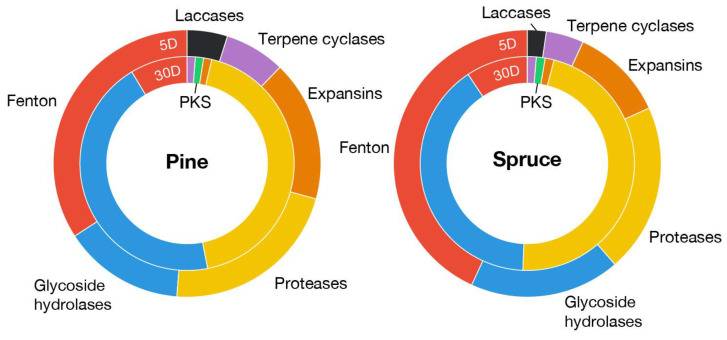
Differentially expressed genes of selected functions relevant to wood decay. The labels ‘5D’ and ‘30D’ indicate samples collected at 5 days (submerged wood) and 30 days (wood wafers), respectively.

The Fenton chemistry is a primary contributor to early wood decay by generating degradative hydroxyl radicals [1]. Among the 69 potential genes involved in Fenton reaction (Supplementary Data S1) in the *F. radiculosa* genome, 20 genes were overexpressed in 5-day submerged samples, including genes encoding quinone reductases (CAZy: AA6), phenol mono-oxygenases (EC:1.14.13.7), aromatic ring mono-oxygenases, phenylalanine ammonia-lyases, glycopeptides, aryl-alcohol oxidases (CAZy: AA3_2), alcohol oxidases (CAZy: AA3_3), glyoxal oxidases (CAZy: AA5_1), amino acid/amine oxidases, iron permeases (Pfam: PF03239), ferroxidases (CAZy: AA1_2), and oxalate decarboxylases (Figure 2). In particular, the genes encoding a glycopeptide (Fibra1|4823), oxalate decarboxylase (Fibra1|666), and alcohol oxidase (Fibra1|657) were among the top 20 most highly up-regulated genes.

In the *F. radiculosa* genome, 18 genes are related to the biosynthesis of Fe^3+^-reducing methoxyhydroquinone, including genes encoding benzoquinone reductases (CAZy: AA6), phenylalanine ammonia-lyases (Pfam: PF00221), aromatic ring mono-oxygenases, phenol mono-oxygenases (EC:1.14.13.7), and O-methyltransferases (EC:2.1.1.64). Except for O-methyltransferase-encoding genes, at least one copy of these genes was overexpressed in submerged ground wood, while none were in overexpressed wood wafers. In addition, the genome contained ten genes encoding glycopeptides, and half of the genes were overexpressed in the ground wood, while one was overexpressed in wood wafers (Supplementary Data S1).

**Figure 3:**
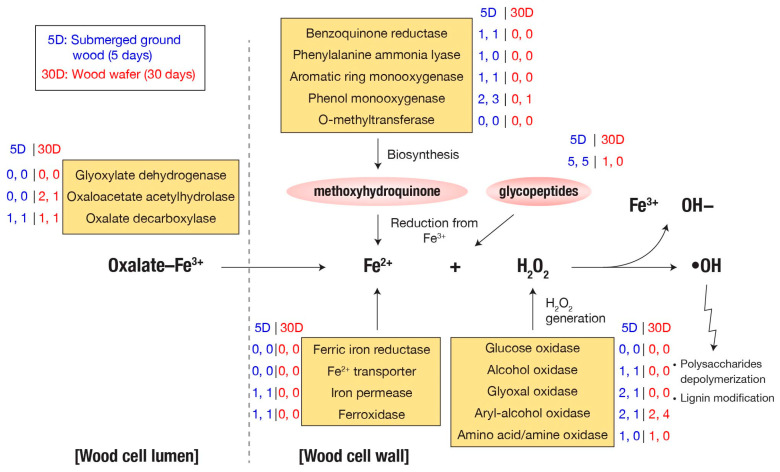
Fenton reaction genes. The values next to each gene name are the number of differentially expressed genes. Two values separated by a comma indicate spruce and pine samples, respectively.

H_2_O_2_ molecules, reacting with Fe^2+^ to generate hydroxyl radicals in the Fenton reaction (Figure 3), can be produced by various secreted oxidases. Except for glucose oxidase-encoding genes, at least one gene copy of each of the following oxidases was overexpressed in submerged ground wood: alcohol oxidase (CAZy: AA3_3), glyoxal oxidase (CAZy: AA5_1), aryl-alcohol oxidase (CAZy: AA3_2), and amino acid/amine oxidase [1].

Oxalate, essential in brown-rot decay by either binding to Fe^2+^ or mobilizing Fe^3+^ [10], can be synthesized from various precursors by glyoxylate dehydrogenase, oxaloacetate acetylhydrolase, and isocitrate lyase [10]. None of the genes that encode oxalate synthesis were up-regulated in submerged ground wood, but two genes encoding oxaloacetate acetylhydrolases (Fibra1|9134 and Fibra1|6892) were up-regulated in the wood wafers. On the other hand, two genes encoding oxalate decarboxylases, which degrade oxalate, are significantly up-regulated in submerged ground wood (Fibra1|666; 316-fold increase in expression) and wood wafer (Fibra1|7873; 315-fold increase). Also, their fragments per kilobase of transcript per million mapped reads (FPKM) values are ranked 16th and 148th, respectively, among all predicted genes. These results corroborate the view that fine control of oxalate level outside of hyphae is affected by extracellular oxaloacetate acetylhydrolase.

### 3.3. Aryl-Alcohol Oxidoreductase for Quinone Metabolism

**Figure 4:**
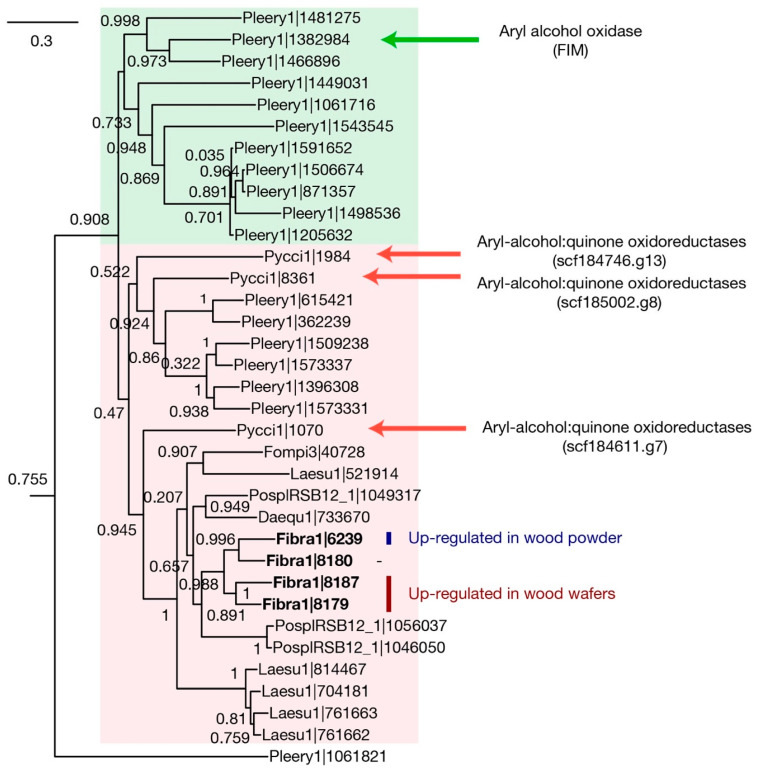
A gene tree of AA3_2 (glucose-methanol-choline oxidoreductases family subfamily 2). The genes were retrieved from five brown-rot fungi and two white-rot fungi, namely *Pycnoporus cinnabarinus* (Pycci1) and *Pleurotus eryngii* (Pleery1). The tree only shows the AAO—AADH group, one of the eight AA3_2 subfamilies, for simplicity. Four experimentally characterized genes are indicated. Each MycoCosm portal ID (http://mycocosm.jgi.doe.gov/, accessed on 8 August 2019) represents a genome as follows: Daequ1, *Daedalea quercina*; Fibra1, *Fibroporia radiculosa*; Fompi3, *Fomitopsis pinicola*; Laesu1, *Laetiporus sulphureus*; PosplRSB12_1, *Postia placenta*.

The AA3_2 family consists of eight phylogenetic subgroups, of which one contains aryl-alcohol oxidases (AAO) and aryl-alcohol dehydrogenases (AADH). These two enzymes generate H_2_O_2_ and hydroquinone, respectively [23,24]. We built a gene tree of the AA3_2 family from the six brown-rot Polyporales fungi. All AAO—AADH genes were predicted to be extracellular, although more than half (58%) of the entire family was intracellular. The six genomes had 8–26 AA3_2 genes, and among them, 1–5 genes belonged to the AAO—AADH group (Figure 4). The *F. radiculosa* genome has four genes belonging to the AAO—AADH group, of which one (Fibra1|6239) was up-regulated in submerged ground wood 24–48-fold, while the other two (Fibra1|8187 and Fibra1|8179) were up-regulated in wood wafers 4–23-fold.

In the gene tree, the genes encoding AAOs and AADHs are separated as two groups, and brown-rot AA3_2 genes are closer to AADH (Figure 4). The gene that was up-regulated in submerged ground wood may be involved in quinone redox cycling by generating hydroquinone, which is required for the Fenton reaction. The other two genes up-regulated in wood wafers could have different biological functions. It has also been suggested that AAOs reduce phenoxy radicals generated via lignin degradation to prevent the re-polymerization of lignin [24]. Since brown-rot only modifies but does not degrade lignin, those AAOs might have a role in keeping the modified state of lignin.

### 3.4. Other Genes Up-Regulated in Submerged Ground Wood

The genes encoding expansins, laccases, and terpene cyclases were up-regulated in submerged ground wood, along with the genes involved in Fenton reaction (Figure 2). In *F. radiculosa*, seven genes encoding expansins were up-regulated, while one was down-regulated. In particular, two expansin genes (Fibra1|7880 and Fibra1|7687) were >500-fold up-regulated and ranked as the 2nd and 25th most expressed genes in submerged ground wood, respectively. “Plant cell wall-loosening” expansin-like proteins in fungi bind to polysaccharides such as cellulose, xylan, and chitin to enhance the accessibility of decay enzymes (e.g., cellulases and xylanases) [25,26]. The genes encoding expansins have been reported to be co-expressed with the genes involved in Fenton reaction in early stages of wood decay in two independent studies of *P. placenta*, where two genes encoding expansin-like proteins were up-regulated in the hyphal front [9] and on wood treated with furfuryl alcohol [27]. This result suggests that expansins might coordinate with the Fenton hydroxyl radicals to enhance the attack by decay enzymes, such as GHs and CEs, in later decay.

There are two genes encoding laccases (CAZy: AA1_1) in the *F. radiculosa* genome, both of which are up-regulated in submerged ground wood (2 to 208-fold). It is hypothesized that laccases play a role in lignin degradation by oxidizing the hydroquinones that generate hydroxyl radicals [4]. It was experimentally supported by 2,5-DMHQ found in decaying wood and potentially oxidized by laccase of *P. placenta* [28]. The up-regulation of genes encoding laccases is consistent with the up-regulation of genes encoding hydroquinone biosynthesis enzymes (Figure 3), where laccases and hydroquinones might coordinate during the substrate decay. In a previous study of *P. placenta*, laccase genes were not differentially expressed between cellulose and glucose in the culture of five days [4]. As the genes involved in Fenton reaction were up-regulated in cellulose in that study, other factors, such as non-cellulosic wood components, could activate laccase genes.

Three terpene cyclase genes were up-regulated in submerged ground wood, while one was up-regulated in wood wafers. In particular, a terpene synthase gene (Fibra1|4870) was up-regulated ~1000-fold in submerged ground wood. Many terpene molecules have antimicrobial activity [29]. The up-regulation of these genes might indicate that the fungi compete with other organisms when they start growing.

Several glycoside hydrolase gene families were exclusively up-regulated in submerged ground wood: GH16, GH37, and GH13, although most GHs were up-regulated in wood wafers. These glycoside hydrolases may be responsible for consuming early available carbon sources. Though it is a smaller number than in wood wafers, 11 protease genes were up-regulated in submerged ground wood.

### 3.5. Genes Encoding Plant Cell Wall Degrading Enzymes

The transcriptome of the 30-day wood wafer culture showed the up-regulation of various plant cell-wall-degrading enzyme genes. It includes glycoside hydrolase (GH), carbohydrate esterase (CE), peptidase, and cytochrome P450 genes. We observed that 40 and 38 GH and peptidase genes, respectively, were up-regulated in either pine or spruce, while 10 and 13 were down-regulated (Figure 2). The up-regulated genes encode cellulases (e.g., GH3, GH45, and GH5_5), hemicellulases (e.g., GH5_7, GH10, CE16), and pectinases (e.g., GH28, CE8, GH53, and GH78). The copper-dependent lytic polysaccharide mono-oxygenases, originating from the AA9 and AA14 families, acting on cellulose and xylan, respectively, were also up-regulated (5- to 34-folds).

One dye-decolorizing peroxidase (DyP) gene (TIGRFAMs: TIGR01413, Fibra1|2048) was also up-regulated about 5-fold in both pine and spruce. It has been suggested that DyPs are the main ligninolytic enzymes of the white-rot fungus *Irpex lacteus* in a lignin-supplemented medium [30]. The enzyme shows peroxidase activity in the presence of H_2_O_2_. It is unexpected because H_2_O_2_ generation is related to the Fenton reaction, and many involved genes were down-regulated in wood wafers. However, we also observed that two aryl-alcohol oxidase genes (CAZy: AA3_2, Fibra1|8187, Fibra1|1982) are up-regulated in wood wafers and may work with DyPs. Among the six brown-rot fungi, only *F. radiculosa* and *P. placenta* genomes contain DyP genes with one or two copies. This result is in contrast to genomes of white-rot fungi, where this gene family is expanded [2]. Due to the lack of conservation, DyP may not play an essential role in brown-rot; the advantage that this gene can bring during wood decay remains unclear.

Another notable differentially expressed group of genes was a polyketide synthase (PKS) gene cluster consisting of PKS, transporter, and cytochrome P450 genes (Fibra1|8597, Fibra1|8600, Fibra1|8602, and Fibra1|8603) that were up-regulated 9.8-, 8.0-, 17.1-, and 42.2-fold in wood wafers, respectively. Many fungal polyketide molecules show antimicrobial activity [31]. As the fungus was grown in a sterile condition, it is unlikely to be activated in response to another microorganism. Also, it is not clear why this gene cluster was down-regulated in submerged ground wood.

### 3.6. Expanded Gene Families and Their Up-Regulation in Wood Wafers

There were 112 expanded and 21 contracted gene families in the *F. radiculosa* genome compared to the five brown-rot relatives (Supplementary Table S3 for the genome list). We identified that 58.0% of the expanded gene families encode secreted proteins. A gene family unique to *F. radiculosa* was OG0000006 (Supplementary Data S2 and S3), which contained 79 genes total, absent in the other five species. The gene family was predicted to be secreted and have a leucine-rich repeat domain (Pfam: PF07723). The leucine-rich repeat domains are involved in protein–protein interactions [32]. The closest homolog was a hypothetical gene in the *P. placenta* genome, one of the five relatives, with around 13% sequence identity. We infer that this gene family was present in the common ancestor of *F. radiculosa* and *P. placenta*, before being duplicated in each species and diverged into separate gene families. In total, 17 and 13 genes of OG0000006 were up-regulated in spruce and pine wood wafers, respectively, suggesting that they may work in concert with carbohydrate-active enzymes that are up-regulated in wood wafers.

**Figure 5:**
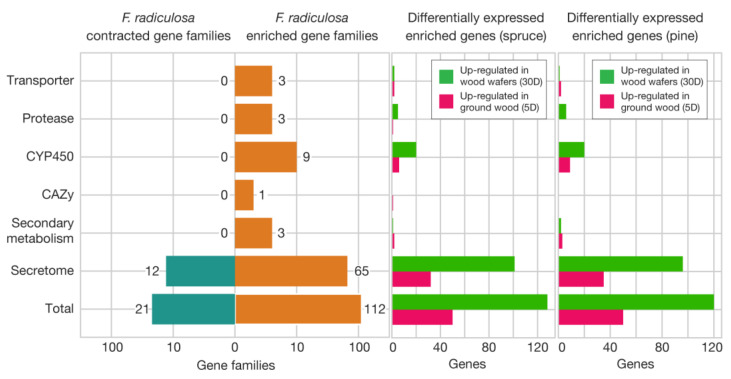
Expanded gene families in *F. radiculosa* over five other brown-rot fungi. Fisher’s exact tests were performed for each gene family (*p* < 0.01). The genes in the expanded gene families were further investigated to determine whether they are up- or down-regulated in ground wood and wafer samples for spruce and pine.

Other gene families expanded in the *F. radiculosa* genome include three transporters, three proteases, nine cytochrome P450, one oxidoreductase, one terpene synthase, and one polyketide synthase families (Figure 5, Supplementary Data S2). We identified that more expanded gene families are up-regulated in wood wafer samples than submerged ground wood samples (Figure 5). For example, 128 genes in expanded gene families were up-regulated in spruce wood wafers, while 50 genes were up-regulated in spruce submerged ground wood. The pattern is most notable in protease and cytochrome P450 gene families. One secreted protease family, namely OG0000125, contains 12 copies in the *F. radiculosa* genome, compared to 0–5 copies in other wood decay fungi. Of these 12 copies in *F. radiculosa*, 3 were up-regulated in wood wafers. Similarly, 15 genes of the 3 expanded cytochrome P450 families were up-regulated in wood wafers. 

### 3.7. Comparative Transcriptomic Trends in F. radiculosa and P. placenta

It has been suggested that the brown-rot fungus *P. placenta* implements two transcriptionally regulated decay modes: early oxidative pretreatment and late hydrolytic saccharification [9]. We observed a similar pattern in our results when comparing *F. radiculosa* transcriptomes from growth on 5-day submerged ground wood and 30-day wood wafers. For example, similar to the 15 up-regulated genes involved in Fenton reaction in the early decay of *P. placenta* [9], *F. radiculosa* shows that 11 orthologs to those genes were up-regulated in submerged ground wood (Supplementary Tables S4 and S5). The up-regulated genes included essential genes involved in the Fenton reaction, such as alcohol/glucose oxidases and benzoquinone reductases. Also, expansin and laccase genes were up-regulated in both the early decay of *P. placenta* and submerged ground wood of *F. radiculosa*. The observed similarity between the wood wafer condition in *F. radiculosa* and the late decay phase in *P. placenta* is somewhat expected. This is because both *F. radiculosa* and *P. placenta* were grown on wafers for extensive periods of time (30 days for *F. radiculosa* and 12–14 days for *P. placenta*). However, it is intriguing that transcriptomic patterns formed by submerged ground wood and early decay were similar.

Submerged ground wood medium is characterized by the breakdown of wood structure and release of easily accessible simple sugars. The report on *P. placenta* [9] suggested that the early decay mode lasted for only two days. As we cultivated *F. radiculosa* for five days on submerged ground wood, it may be that the mycelia consumed the simple sugars first and had started to degrade the remaining polysaccharides by the time that we isolated RNA. In another report on *P. placenta* [4], the genes involved in Fenton reaction were up-regulated, with cellulose being the sole carbon source compared to glucose. Overall, the genes involved in Fenton reaction seem to be activated by recognizing polysaccharides, regardless of their physical state (ground wood or wafers), and the decay mode subsequently advances to the hydrolytic mode of decay.

## 4. Conclusions

Our study explored the transcriptome of the brown-rot fungus *Fibroporia radiculosa*, with a focus on assessing how wood species and culture conditions influenced gene expression. We found that while wood species had a limited impact, the conditions of ground wood versus wood wafers had a significant influence on the transcriptome profile. These findings offer valuable insights into the mechanisms underlying plant biomass conversion by this fungal species. Given the independent evolution of brown-rot fungi on multiple occasions, investigating the effects of wood species and conditions on the growth and gene expression patterns of other species would be instrumental to determining the conservation of these observed patterns among brown-rot fungi.

## Data Availability

We used the previously reported genome assembly, gene predictions, and annotations of *Fibroporia radiculosa* TFFH 294 [12], and they are available in the MycoCosm [33] (https://mycocosm.jgi.doe.gov/Fibroporia_radiculosa, accessed on 1 April 2019). The transcriptome data are available at NCBI Sequence Read Archive (https://www.ncbi.nlm.nih.gov/sra, accessed on 9 October 2018) with accessions of SRP164803–164817.

## Supplementary Materials

The following supporting information can be downloaded via this link: https://www.mdpi.com/article/10.3390/jof9101029/s1, Figure S1. Pearson’s correlation between samples; Table S1. Reads alignments; Table S2. Annotation of genes involved in Fenton reaction; Table S3. Brown-rot fungal genomes for comparative analysis; Table S4. The genes involved in Fenton reaction up-regulated during early decay reported in *Postia placenta*, and their orthologs were also up-regulated in submerged ground wood in *Fibroporia radiculosa*; Table S5. The genes encoding plant cell wall-degrading enzymes up-regulated in late decay were reported in *Postia placenta*, and their orthologs were also up-regulated in wood wafers in *Fibroporia radiculosa*; Data S1. Differential gene expression of *Fibroporia radiculosa*; Data S2. Expanded gene families in *F. radiculosa* over five other brown-rot fungi; Data S3. Orthologous groups of *F. radiculosa* and five brown-rot relatives.
