# Supplementary material for: Transcriptomics of Temporal- versus Substrate-Specific Wood Decay in the Brown-Rot Fungus Fibroporia radiculosa
Source: J Fungi (Basel). 2023 Oct 19;9(10):1029. doi: 10.3390/jof9101029 (PMC10608345; doi:10.3390/jof9101029)
Supplement: Supplementary file 1 [file jof-09-01029-s001.zip › Fibra transcriptome Supp Figures and Tables v1.9.pdf]

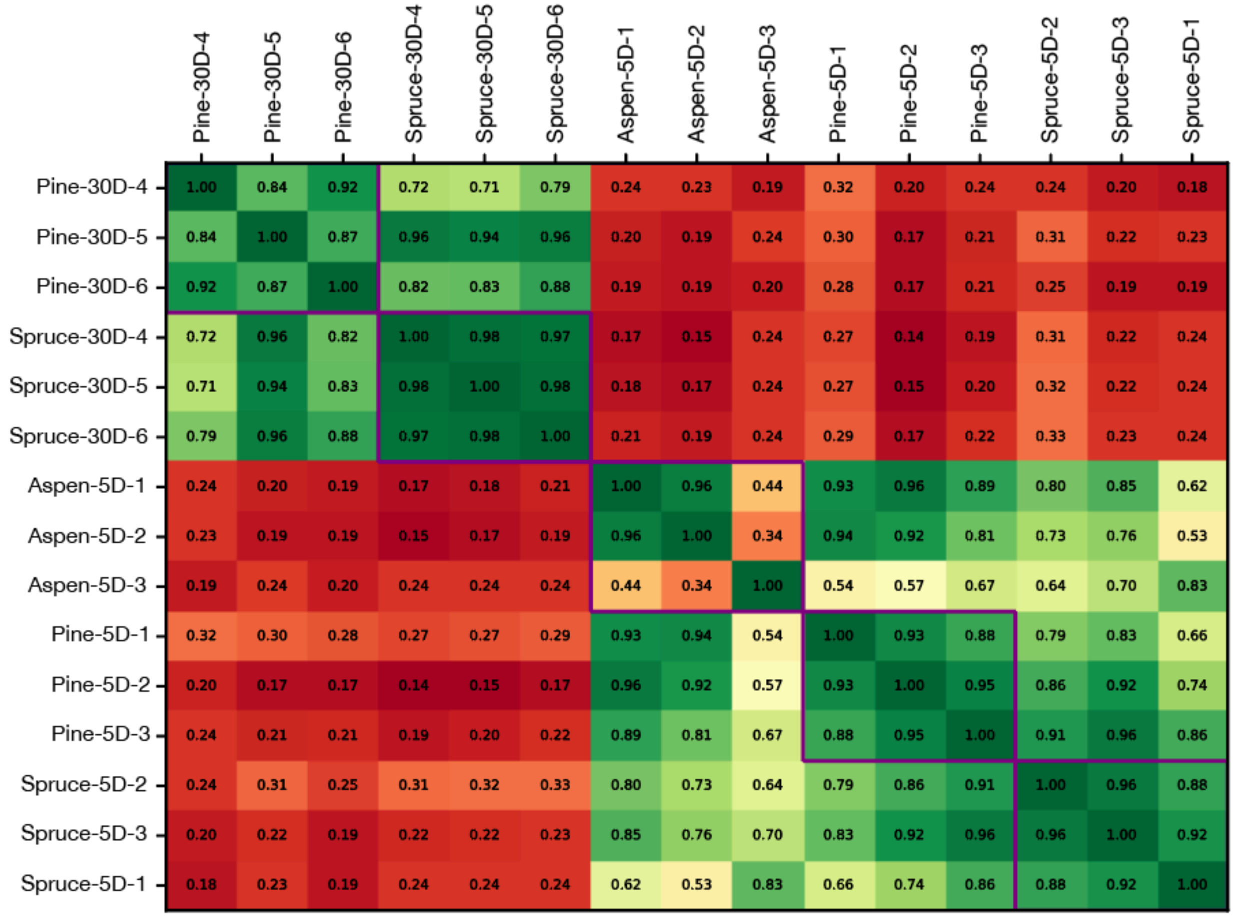

Supplementary Figure S1. Pearson's correlation between samples. Raw read counts were used as input for the calculation.

Supplementary Table S1. Reads alignments.

Library	Filtered reads	Mapped reads	Mapping rate
Pine-30D-4	10,907,540	9,914,794	90.90%
Pine-30D-5	12,340,565	11,330,711	91.82%
Pine-30D-6	12,073,619	10,942,616	90.63%
Spruce-30D-6	16,813,935	15,302,579	91.01%
Spruce-30D-5	16,628,360	15,147,650	91.10%
Spruce-30D-5	12,821,901	11,548,856	90.07%
Aspen-5D-1	17,252,636	13,652,388	79.13%
Aspen-5D-2	19,275,033	17,453,461	90.55%
Aspen-5D-3	18,703,720	14,026,912	75.00%
Pine-5D-1	17,455,078	13,796,245	79.04%
Pine-5D-2	16,634,692	14,257,903	85.71%
Pine-5D-3	19,833,307	16,220,407	81.78%
Spruce-5D-2	15,978,872	13,239,527	82.86%
Spruce-5D-3	16,299,746	13,490,015	82.76%
Spruce-5D-1	14,994,961	10,939,960	72.96%

Supplementary Table S2. Annotation of genes involved in Fenton reaction. We define orthologs as members of Markov clustering and homologs as BLASTp hits (E value cutoff 1E-5). The CAZy, Enzyme Commission (EC), and Pfam annotations are available in the MycoCosm (https://mycocosm.jgi.doe.gov/).

Pathway	Name	Annotation
Quinone redox cycling and hydroquinone biosynthesis	Quinone reductase	CAZy:AA6
	Phenol monooxygenase	EC:1.14.13.7
	Aromatic ring monooxygenase	Orthologs of Pospl1|54109, Pospl1|23052, and Pospl1|129128
	Phenylalanine ammonia-lyase	Pfam:PF00221
	O-methyltransferase	EC:2.1.1.64
Fe^{3+} reduction to Fe^{2+}	Glycopeptides	Homologs of Phchr2|3023986 (glp1) and Phchr2|3023982 (glp2)
H_2O_2 generation	Aryl-alcohol oxidase	CAZy:AA3_2*
	Alcohol oxidase	CAZy:AA3_3
	Glyoxal oxidase<br>Copper radical oxidases	CAZy:AA5_1<br>CAZy:AA5_2
	Amino acid<br>Amine oxidases	Orthologs of<br>Pospl1|47008 and Pospl1|110689
Iron reduction and homeostasis genes	Ferric iron reductase	Pfam:PF08030,PF01794
	Iron permease	Pfam:PF03239
	Fe^{2+} transporter	Orthologs of<br>Pospl1|52765 and Pospl1|43266
	Ferroxidase	CAZy:AA1_2
Oxalate metabolism	Glyoxylate dehydrogenase	Orthologs of Pospl1|121561 and Pospl1|115965
	Oxaloacetate acetylhydrolase	Orthologs of Pospl1|112832
	Oxalate decarboxylase	Orthologs of Pospl1|46778

*AA3_2 family has eight subgroups of which one subgroup has aryl-alcohol oxidase activity. See the methods in the main text.

Supplementary Table S3. Brown rot fungal genomes for comparative analysis. Each genome portal can be accessible via https://mycocosm.jgi.doe.gov/**[PORTAL_ID]**.

Strain	Portal ID in MycoCosm	Genome paper (Pubmed ID)
Daedalea quercina	Daequ1	26659563
Fomitopsis pinicola FP-58527 SS1	Fompi3	22745431
Laetiporus sulphureus var. *sulphureus*	Laesu1	26659563
Postia placenta MAD-698-R-SB12	PosplRSB12_1	28831381
Wolfiporia cocos MD-104 SS10	Wolco1	22745431

Supplementary Table S4. The genes involved in Fenton reaction up-regulated during early decay reported in *Postia placenta*, and their orthologs also up-regulated in submerged ground wood in *Fibroporia radiculosa*. The genes with a false discovery rate <0.05 and fold change >4 are assigned as differentially expressed (same as performed in the *P. placenta* study).

**Gene family**	***P. placenta* in early decay (reported by Zhang et al.)**	***F. radiculosa* in ground wood (observed in this study)**
Glucose oxidase<br>Alcohol oxidase	44331<br>128830<br>55972<br>129158/126217	657<br>3364<br>6239
Glyoxal oxidase	46390	5062**
Amino acid/amine oxidase	47008	2469*
Amino acid/amine oxidase	110689	1927
Iron reductase	130025	2484*
Iron reductase	130030/130043	-
Fe^{2+} transporters	52765/43266	-
Benzoquinone reductase	124517/64069	8914
Phenylalanine ammonia-lyase	111514	6596
Aromatic ring monooxygenases	54109/46071<br>23052/62058<br>129128	1441<br>4445

*They are differentially expressed (false discovery rate <0.05), but have fold change values from two to four.

**The genes are not in the same gene family, but they are annotated having the same function and are reciprocal best hit in the BLAST alignment.

Supplementary Table S5. The genes encoding plant cell wall-degrading enzymes up-regulated in late decay reported in *Postia placenta*, and their orthologs also up-regulated in wood wafers in *Fibroporia radiculosa*. The genes with a false discovery rate <0.05 and fold change >4 are assigned as differentially expressed (same as performed in the *P. placenta* study).

Gene family	***P. placenta*** **in late decay (reported by Zhang et al.)**	***F. radiculosa*** **in 30 days (observed in this study)**
Endoglucanases Cel5A<br>Endoglucanases Cel5B	115648/108962<br>103675/117690	3096<br>3097<br>4239
Endoglucanases Cel12A	121191/112658	9027<br>3175
Xylanase Xyn10A-1	113670/90657	1548<br>1546*<br>1549*
Mannanase Man5A	57321/121831	1048<br>2871

*They are differentially expressed (false discovery rate <0.05), but have lower fold change values from two to four.
